# Evaluation of Factors Influencing Liver Function Test in On-Pump Coronary Artery Bypass Graft Surgery

**Published:** 2013-12

**Authors:** Shahrbano Shahbazi, Ashkan Panah, Mohammad Ali Sahmeddini

**Affiliations:** Shiraz Anesthesiology and Intensive Care Research Center, Nemazee Hospital, Shiraz University of Medical Sciences, Shiraz, Iran

**Keywords:** Liver function tests, Cardiopulmonary bypass, Coronary artery bypass

## Abstract

**Background:** Liver dysfunction during on-pump coronary artery bypass graft surgery (CABG) is a rare complication but is associated with significant morbidity and mortality. The ability to identify high-risk patients may be helpful in planning appropriate management strategies. We aimed to evaluate the factors influencing liver function tests during on-pump CABG.

**Methods: **In 146 patients scheduled for on-pump CABG, the liver function test was done preoperatively and on the first postoperative day. Some preoperative and intraoperative risk factors were checked and then the postoperative liver function tests were compared with the preoperative ones. Probable relationships between these changes and the preoperative and intraoperative risk factors were studied.

**Results: **A medical history of diabetes had a significant relationship with the changes in direct bilirubin. Preoperative central venous pressure had a significant relationship with the changes in aspartate aminotransferase and alanine aminotransferase. Use of intra-aortic balloon pump and duration of aortic cross-clamp were significantly related to the changes in the liver function tests except for alanine aminotransferase and alkaline phosphatase.

**Conclusion:** It seems that the techniques for the reduction of cardiopulmonary bypass and aortic cross-clamp duration may be useful to protect liver function. We recommend that a larger population of patients be studied to confirm these findings.

## Introduction

In coronary artery bypass graft surgery (CABG), various changes take place in the different organs of the body because of the effect of bypass pumps and hemodilution.^1^ The liver is one of the most vital organs and is highly prone to damage during CABG. Dilutional anemia and hemodynamic changes can affect tissue oxygenation, and most studies state that a minimum hematocrit level of 22% is necessary for the on-pump technique.^2^

Suitable perfusion and tissue oxygenation is considerably effective in the function of organs before, during, and after surgery.^3^ During cardiopulmonary bypass (CPB), the possibility of liver damage increases owing to the-non pulsatile perfusion, low-flow state, free radicals formation, and increased levels of catecholamines.^4^ Some studies have reported that CPB usually induces mild hepatocellular damage, whereas off-pump coronary bypass decreases the possibility of this damage.^5^ However, reports on CPB are conflicting because hypothermia decreases the oxygen demand of the splanchnic organs and, thus, hepatocellular oxygenation is preserved better during hypothermic CPB.^6^ Comparison between pulsatile and non-pulsatile flows during CPB shows no significant difference between the two flows during CPB.^7^ Moreover, comparison between CABG with or without CPB demonstrates that the liver metabolic function is not changed by the type of coronary bypass surgery but that hepatic ischemia is detected after cardiac surgery with CPB, which is usually marked with an increase in alanine aminotransferase (ALT) enzyme levels.^8^

Given the inconsistency in the studies on the effects of CPB on the liver function test post on-pump CABG, we aimed to evaluate the impact of CPB and other factors that may aggravate changes on the liver function test after on-pump cardiac surgery. 

## Patients and Methods

In this quasi-experimental clinical trial, which was done during 2011, after obtaining approval from the Ethics Committee and written informed consent from the patients, 146 out of 190 patients who referred to hospitals affiliated to Shiraz University of Medical Sciences, southern Iran, for elective CABG were recruited. The sample size was calculated to be 142 patients considering α of 0.05, power of 80%, and standard deviation (SD) of 9 (using the power static software calculator [SSC]). 

Patients who had simultaneous cardiac valvular surgery, those with hemolytic disorders, and those with abnormal liver function tests prior to surgery or those with a history of fatty liver or chronic liver disease were excluded from the study.

The patients’ data including age, sex, Body Surface Area, hematocrit level, direct and indirect bilirubin levels, hepatic enzymes (aspartate aminotransferase [AST], ALT, and alkaline phosphatase [ALP]), serum creatinine, ejection fraction, history of cardiovascular and cerebral diseases, and history of diabetes mellitus were recorded in a specific form.

Following anesthesia induction using Midazolam, Sufentanil, Na-Thiopental, and Pancuronium, Morphine was administered for the patients. After inserting the central venous catheter in place, the initial central venous pressure (CVP) was recorded. Oxygen and Isoflurane were used for the maintenance of anesthesia.

A CPB pump with a flow of 2.4-2.6 lit/min/m^2^ and a temperature of at least 32°C was used. Anesthesia was maintained using the α-stat method for arterial blood gas management, and the mean arterial blood pressure was kept at 60-70 mm Hg. Upon necessity, the patients’ blood pressure was maintained using inotrope and vasopressors. Moreover, in order to correct the hematocrit level, blood transfusion was done for the patients if needed.

At the end of surgery, the patients’ minimum hematocrit level, number of infused blood packs, use of intra-aortic balloon pump, CPB time, aortic cross-clamp time, and use of inotrope and vasopressors until the first postoperative day were recorded. After the transfer of the patients to the Intensive Care Unit (ICU), data regarding serum bilirubin (direct and indirect), ALT, AST, and ALP for the first postoperative day were also recorded. 

The data were analyzed using SPSS software (version 16). In order to determine the normality of the data, the Kolmogorov-Smirnov test was utilized. The paired *t *test, analysis of variance (ANOVA), and the Pearson correlation coefficient were employed as appropriated and multiple regression test was used for adjustment. A P<0.05 was considered statistically significant. 

## Results

Eighty-six (58.9%) patients were men, 41 (28.1%) patients had a history of myocardial infarction, and 53 (36.3%) patients had a history of diabetes mellitus. Intra-aortic balloon pumps were not used for 132 (90.4%) patients. Table 1 shows the mean±SD of some of the patients’ quantitative variables.

**Table 1:** Patients’ quantitative variables (mean±SD)

**Variable**	**mean±SD**
Age (years)	65.62±10.47
Body Surface Area (m^2^)	1.68±0.14
Hematocrit (%)	39.45±5.11
Creatinine (mg/dl)	1.12±0.32
Ejection fraction (%)	47.76±10.61
Cardiopulmonary bypass time (min)	77.11±22.57
Cross-clamp duration (min)	43.97±16.01

The mean±SD of direct and indirect bilirubin changes, ALP, ALT, and AST was 0.137±0.45, 0.378±1.19, -45.12±8.2, 11.15±2.88, and 41.46 7.56, respectively. 

Except for ALP, all the other liver function test indices had a significant increase after surgery (table 2). Comparison of the liver function test indices before and after surgery between both sexes demonstrated no significant difference between the men and women in this regard. Also, there was no significant difference between the liver function test results before and after surgery between the patients with and without a history of diabetes, except in their direct bilirubin levels. Moreover, no significant difference was detected between a history of myocardial infarction and changes in the liver function test indices before and after surgery. Except for AST, no significant difference was seen in hepatic enzymes before and after surgery between the patients receiving an intra-aortic balloon pump and those who did not. However, the mean AST change in the patients who received the intra-aortic balloon pump was more than that in the patients who did not (table 3). No significant difference was observed between the number of infused blood packs and changes in the liver function tests before and after surgery (table 4). Furthermore, the changes in the liver enzymes and bilirubin before and after surgery were compared with history of diabetes, history of myocardial infarction, and intra-aortic balloon pump use via the multiple regression test, which revealed no significant relationships (P=0.22 for diabetes, P=0.82 for myocardial infarction, and P=0.27 for intra-aortic balloon pump). 

**Table 2:** Liver function test indices before and after surgery (mean±SD)

**Indices**	**Before surgery**	**After surgery**	**P value**
Direct bilirubin (mg/dl)	0.286±0.355	0.423±0.435	<0.001
Indirect bilirubin (mg/dl)	0.672±0.759	1.05±1.03	<0.001
ALP(U/dl)	210.79±104.66	165.67±67.83	>0.25
ALT(U/dl)	35.26±30.39	46.41±37.72	<0.001
AST(U/dl)	39.53±37.31	80.99±84.43	<0.001

ALP: alkaline phosphatase; ALT: alanine aminotransferase; AST: aspartate aminotransferase

**Table 3:** Relationship between the changes in the liver function tests with respect to sex, history of diabetes, history of myocardial infarction, and use of intra-aortic balloon pump, before and after surgery (mean±SD)

**Indices**	**Sex**			**History of diabetes**			**History of myocardial infarction**			**Intra-aortic balloon pump**		
	**Men**	**Women**	**P value**	**Yes**	**No**	**P value**	**Yes**	**No**	**P value**	**Yes**	**No**	**P value**
Direct bilirubin (mg/dl)	0.133±0.544	0.144±0.279	0.875	0.034±0.467	0.196±0.437	*0.038	0.144±0.684	0.134±0.326	0.930	0.240±0.517	0.126±0.447	0.374
Indirect bilirubin (mg/dl)	0.283±1.32	0.512±0.690	0.254	0.214±1.32	0.470±1.1	0.212	0.324±1.58	0.398±1.0	0.736	0.544±0.428	0.359±1.24	0.582
ALP(U/dl)	-44.88±88.17	-45.57±73.23	0.957	-43.66±72.62	-45.96±87.41	0.865	-53.15±107.97	-41.99±69.84	0.463	-71.36±64.55	-42.34±83.46	0.210
ALT(U/dl)	10.17±31.19	12.55±25.07	0.625	13.53±20.92	9.79±32.42	0.453	10.85±25.75	11.27±29.97	0.938	16.78±25.88	10.55±29.08	0.443
AST(U/dl)	36.86±57.93	48.07±95.69	0.380	38.6±58.16	43.1±84.27	0.731	44.68±50.14	40.21±83.71	0.749	37.19±73.06	81.78±90.11	*0.036

ALP: Alkaline phosphatase; ALT: Alanine aminotransferase; AST: Aspartate aminotransferase; *Significant difference

**Table 4:** Relationship between the liver function tests and the number of infused packed blood cell units, before and after surgery (mean±SD)

**Indices**	**Zero** **packed blood cell units**	**One** **packed blood cell unit**	**Two** **packed blood cell units**	**Three or more** **packed blood cell units**	**P value**
Direct bilirubin (mg/dl)	0.193±0.349	0.149±0.406	0.063±0.562	0.160±0.469	0.542
Indirect bilirubin (mg/dl)	0.413±0.462	0.638±1.14	0.067±1.4	0.732±1.86	0.098
ALP(U/dl)	-50.19±64.86	-24.5±88.97	-58.06±94.42	-26.29±76.54	0.255
ALT(U/dl)	13.33±31.31	7.61±40.49	9.34±19.75	15.53±19.17	0.731
AST(U/dl)	42.21±99.64	29.93±38.85	45.76±70.66	45.82±48.86	0.836

ALP: Alkaline phosphatase; ALT: Alanine aminotransferase; AST: Aspartate aminotransferase

There were no significant differences between the changes in the liver function tests and age, Body Surface Area, hematocrit, and lowest hematocrit level of the patients during surgery (the Pearson correlation coefficient test). The creatinine level showed a reverse significant correlation with the mean indirect bilirubin changes (P<0.05). However, it did not have any correlation with the mean changes in the other hepatic enzymes (P>0.05). The ejection fraction had a significant reverse correlation with the mean ALP changes (P<0.05), and it did not have a significant correlation with the other liver function tests (table 5). 

**Table 5:** Correlation between the liver function tests and the studied quantitative variables, before and after surgery (mean±SD)

**Quantitative variables**	**Correlation (P value)**				
	**Direct bilirubin (mg/dl)**	**Indirect bilirubin (mg/dl)**	**ALP (U/dl)**	**ALT (U/dl)**	**AST (U/dl)**
Age (years)	0.078 (0.352)	0.085 (0.309)	-0.008 (0.922)	-0.003 (0.976)	0.028 (0.739)
BSA (m^2^)	-0.079 (0.341)	-0.150 (0.071)	0.132 (0.113)	0.081 (0.330)	-0.055 (0.510)
Hct (%)	-0.069 (0.406)	-0.052 (0.536)	-0.019 (0.823)	-0.103 (0.218)	-0.070 (0.403)
Cr (mg/dl)	-0.090 (0.281)	-0.168 (0.042)*	-0.057 (0.497)	0.094 (0.259)	-0.02 (0.814)
EF (%)	-0.016 (0.849)	0.0.94 (0.260)	-0.167 (0.044)*	0.036 (0.668)	0.071 (0.392)
CVP (mg/dl)	-0.118 (0.156)	0.041 (0.622)	0.034 (0.684)	0.303 (0.001)*	0.335 (0.001)*
Lowest Hct (%)	0.045 (0.588)	0.012 (0.886)	0.047 (0.571)	-0.039 (0.639)	-0.098 (0.242)
CPB time (min)	0.211 (0.010)*	0.164 (0.048)*	0.057 (0.494)	0.123 (0.138)	0.192 (0.020)*
Cross-clamp Duration (min)	0.074 (0.374)	0.046 (0.582)	-0.104 (0.210)	0.064 (0.443)	0.174 (0.036)*

BSA: Body surface area; Hct: Hematocrit; Cr: Creatinine; EF: Ejection fraction; CVP: Central vein pressure; CPB: Cardiopulmonary bypass; ALP: Alkaline phosphatase; ALT: Alanine aminotransferase; AST: Aspartate aminotransferase; *Significant level

The CVP had a direct and significant relationship with the mean ALT and AST changes (P<0.05). Moreover, CPB time had a direct and significant relationship with the mean direct and indirect bilirubin and AST (P<0.05), whereas it had no significant correlation with the changes in the ALT and ALP levels. The aortic cross-clamp time only showed a direct significant relationship with AST (table 5). 

In this study, all the patients received inotrope and vasopressors until the first postoperative day. None of the patients had a history of cerebral vascular disease.

## Discussion

For all the recent advances in the techniques and modes of anesthesia in on-pump cardiac surgery, liver complications still remain one of the most severe postoperative consequences of cardiac surgery. 

Although it is believed that a lower hemoglobin level in women, small stature, and age-related atherosclerosis can affect the perfusion of visceral organs,^2^ we found no significant relationship between the changes in the liver function tests and age, sex, and Body Surface Area.

During on-pump cardiac surgery, even with mild hypothermia, the basal metabolic rate is decreased. Hypothermia helps the individual to tolerate low hematocrit because of hemodilution. On the other hand, the drop in hematocrit levels and the resulting reduction in perfusion can damage visceral organs, especially when they are compromised by an underlying disease. A large body of research states that with a drop in hematocrit levels to below 17%, the patient will have serious complications; and if the hematocrit level drops below 14%, the patient might be at risk of death.^9^^-^^13^ In our study, the lowest hematocrit level was not below 22%; consequently, we found no significant relationship between the hematocrit level before surgery, lowest hematocrit level on pump, and changes in the liver function tests.

Mc Sweeny et al.^14^ stated that a history of myocardial infarction, revascularization, and ejection fraction below 40% were the independent factors affecting postoperative gastrointestinal complications. Moreover, the authors found that a history of renal failure was effective in the occurrence of gastrointestinal complications. Also, Raman et al.^15^ reported that a history of diabetes mellitus and heart failure could cause severe liver ischemia after cardiac surgeries. In our study, a history of diabetes had a significant relationship with direct bilirubin changes, and not with the other liver function tests. Also, myocardial infarction and preoperative creatinine levels did not have a significant relationship with any of the liver function tests, while the ejection fraction had a reverse significant relationship with the changes in the ALP levels and the CVP pressure had a direct and significant relationship with the changes in the liver enzymes.

The results of the present study are not in agreement with that of in some previous studies. Conflicting results can also be found between the previous investigations; this is because different studies employ different markers for detecting hepatocellular injury such as alcohol dehydrogenase (AD) and glutathione S-transferase (GST). It is worthy of note that we used conventional transaminases, which are, albeit a lesser indicator of hepatic damage, more practical than the others.

Holmes showed that using inotrope and vasopressors could increase visceral vascular resistance and cause ischemia by vascular contracture. For instance, while infusion of epinephrine could increase cardiac output, it would reduce visceral perfusion.^16^ In our study, inotrope and vasopressors were infused for all the patients until the first postoperative day. Therefore, we could not assess their effect on the changes in the liver function tests.

Some researchers have reported that the use of intra-aortic balloon pumps could reduce tissue perfusion and cause gastrointestinal complications, especially liver complications.^8^ In our study, only AST had a considerably significant increase in the patients for whom intra-aortic balloon pumps were used. Another study showed that inadequate venous drainage caused liver congestion and increased postoperative liver damage. Venous drainage during CPB depends on multiple factors.^17^ However, during CPB, the amount of the pump flow directly correlates with the amount of systemic venous return. In our study, the pump flow was maintained at 2.4-2.6 lit/min/m^2^, so venous drainage during our study was acceptable and did not damage the liver function.

The effects of pump time and aortic cross-clamp time on visceral perfusion have also been studied. Murphy et al.^18^ found that prolong pump time would affect visceral vascular and hepatocellular perfusion. In our study, the CPB time had a direct and significant relationship with the changes in the direct and indirect bilirubin and AST levels. Nevertheless, the aortic cross-clamp time only had a direct and significant relationship with the AST changes, which could indicate the skillfulness of the surgeon in minimizing the bypass time to reduce postoperative complications.

## Conclusion

It seems that the techniques for the reduction of CBP and aortic cross-clamp duration may be useful to protect the liver function. We recommend that future studies be conducted on a larger population of patients and with a single surgeon so as to achieve more comprehensive results.
